# Extracorporeal shock wave therapy for equine musculoskeletal disorders: from biological mechanisms to clinical applications

**DOI:** 10.3389/fvets.2025.1719123

**Published:** 2025-12-19

**Authors:** Zhongsheng Qiu, Jiaqi Wang, Yukun Zhang, Xiaxin Liu, Chengwei Wei, Tianwen Ma

**Affiliations:** Heilongjiang Key Laboratory for Laboratory Animals and Comparative Medicine, College of Veterinary Medicine, Northeast Agriculture University, Harbin, China

**Keywords:** biological mechanisms, clinical application, equine musculoskeletal injuries, extracorporeal shock wave therapy, horse

## Abstract

Musculoskeletal injuries represent a primary cause of suboptimal performance and early retirement in equine athletes. To address this challenge, the veterinary community has long endeavored to develop safer and more effective therapeutic strategies. Extracorporeal shock wave therapy (ESWT), as a treatment for equine musculoskeletal injuries, has garnered substantial attention among equine veterinarians. Focused on the theme ESWT Therapy for Equine Musculoskeletal Disorders: From biological mechanisms to clinical applications, this article systematically reviews existing literature on the biological effects of ESWT—including analgesia, anti-inflammation, and autologous repair—mediated through diverse signaling pathways and factors. It synthesizes the current status of clinical applications and underlying mechanisms of ESWT in managing equine musculoskeletal conditions such as suspensory desmitis, superficial digital flexor tendinitis, osteoarthritis, navicular syndrome, and back pain syndrome. Additionally, the article summarizes relevant parameters for ESWT in treating different injuries, offering a reference for clinical equine veterinarians.

## 1 Introduction

Equines are highly susceptible to musculoskeletal injuries because of their unique physiological characteristics and work demands. Surveys indicate that in low- and middle-income countries, the prevalence of lameness in working equines reaches 29.9%, with gait abnormalities observed in up to 62.9% of animals (1). The high incidence and often refractory nature of these injuries not only result in substantial economic losses but also significantly compromise equine quality of life and welfare. ESWT is a physical modality that delivers shock waves to target tissues via a dedicated generator. ESWT, which was originally developed for human medicine, has the advantages of being noninvasive, relatively low cost, and associated with minimal complications. It has demonstrated promising efficacy in the treatment of various equine musculoskeletal injuries (2–7). However, despite its widespread application in equine musculoskeletal disorders, the mechanisms of action, biological effects, and optimal clinical protocols of ESWT remain insufficiently summarized and explored. Therefore, this review systematically examines the therapeutic mechanisms, number of pulses, treatment sites, and treatment intervals of ESWT. On the basis of the current literature and clinical practice, this study also provides recommended treatment parameters for different types of equine musculoskeletal injuries, with the aim of facilitating the standardization and optimization of ESWT in equine medicine.

### 1.1 ESWT overview

Shock waves (SWs) are mechanical energy waves generated by the rapid compression and aggregation of a medium caused by vibration or high-velocity motion (8). SWs are classified as focused shock waves (piezoelectric, electromagnetic, or electrohydraulic) or radial shock waves (ballistic) on the basis of their focusing characteristics.

On the basis of their energy flux density, shock waves are classified as high-energy (>0.5 mJ/mm^2^), medium-energy (0.3–0.5 mJ/mm^2^), or low-energy (<0.3 mJ/mm^2^), although the threshold values vary slightly among publications (9–11). During propagation, SWs produce abrupt changes in the pressure, temperature, and density of the medium, resulting in a series of physical effects. For example, the cavitation and mechanical actions generated by Extracorporeal shock wave (ESW)promote vascular recanalization, enhance local microcirculation, and disrupt soft-tissue adhesions (12–14). With ongoing rese200h, ESWT has been increasingly applied in human medicine for the treatment and rehabilitation of ligament disorders, joint diseases, delayed bone healing, nerve injuries, and soft-tissue injuries (15).

In equine clinical practice, ESWT is primarily used to treat musculoskeletal disorders such as ligament injuries (2, 3, 16), tendinopathies (6, 17), back pain (7, 18), and osteoarthritis (4, 17, 19). During ESWT performed in clinical practice settings, horses are typically given mild sedation to prevent excessive movement. A portable ESW device that delivers high-pressure acoustic waves is applied to the affected site (bone, joint, tendon, or ligament) for approximately 3–4 min. In most cases, a typical treatment protocol consists of three sessions administered at three-week intervals. Because ESWT can cause temporary desensitization of the treated region after application, concerns have been raised that horses may sustain more severe injuries if they are exercised or made to compete during the analgesic period. Riders have also expressed apprehension regarding potential alterations in gait that could affect performance. Consequently, many jurisdictions enacted regulations prohibiting horses from competing for 7–14 days after ESWT, a regulatory measure that merits close attention from practitioners (20).

## 2 Biological effects of ESWT

The mechanical stress generated by shock waves can induce elastic deformation of cell membrane structures, thereby activating specific mechanosensitive ion channels and enabling the transduction of extracellular mechanical signals into intracellular responses (21–23). This process, in turn, elicits multiple biological effects, including analgesia (23, 24), inhibition of inflammation (9, 24–27), and promotion of endogenous tissue repair (21, 28–30).

### 2.1 Pain relief

ESWT is widely used to improve patients’ pain during clinical diagnosis and treatment. At present, the mechanism by which ESWT produces analgesic effects on the body is still unclear. However, research reports on its application in musculoskeletal injuries indicate that, compared with drugs such as corticosteroids and hyaluronic acid, ESWT has better analgesic effects (21). Existing studies have explored its analgesic mechanism from different angles, mainly in terms of the following aspects:

#### 2.1.1 Nerve injury and regeneration

ESW can induce selective injury to large-diameter myelinated fibers and their myelin sheaths, thereby modulating nociceptive signal transmission and producing analgesic effects. Importantly, this neural injury appears to be reversible (22, 23).

In an experimental model in which focused ESWT (0.08 mJ/mm^2^, 4 Hz, 2,000 pulses) was applied to rat hind paw pads, pronounced damage was observed in large-diameter neurons (cell bodies > 30 μm) known to play a key role in pain transmission, followed by rapid axonal regeneration. Such reinnervation may contribute to resensitization of previously desensitized areas and could be one factor underlying pain recurrence (22).

Bolt et al. performed radial ESWT on the palmar digital nerves of horses (0.25 MPa, 240 pulses/min, 2,000 pulses) and observed extensive separation and disruption of the myelin layers in medium- to large-diameter myelinated axons (axon diameter 5–15 μm). In contrast, no changes were noted in small-diameter myelinated axons (axon diameter 1–5 μm) or in nonmyelinated axons. Nerve injury does not involve axons or Schwann cell bodies but is limited to myelin dysfunction (23).

#### 2.1.2 Regulation of neurotransmitters and neuropeptides

Substance P is a key neurotransmitter in pain transmission and is responsible for conveying peripheral injury signals to the central nervous system. After extracorporeal shock waves (electrohydraulic shock wave generator, 1,500 pulses, 0.9 mJ/mm^2^, 1 Hz) were applied to the rabbit femur, the concentration of substance P in the periosteum on the treated side exhibited a biphasic pattern: compared with that in the contralateral limb, it increased significantly at 6 and 24 h after treatment but decreased markedly after 6 weeks. This dynamic change suggests a possible analgesic mechanism of ESWT: early, acute release of substance P may trigger pain shortly after treatment, whereas subsequent depletion may contribute to long-term analgesia. Notably, this process is not accompanied by changes in prostaglandin E₂, indicating that the effect is independent of the inflammatory response. Shock waves may thus inhibit pain transmission by activating large-diameter nerve fibers, forming a three-phase analgesic mode of “stimulation–depletion–regulation” (31).

ESWT can achieve analgesic effects through multiple mechanisms, including but not limited to affecting nerve damage and regeneration, regulating neurotransmitters and neuropeptides, inhibiting inflammation, and promoting tissue repair (see 2.2 and 2.3 for details on inhibiting inflammation and promoting tissue repair).

### 2.2 Inhibition of inflammation

Low-energy ESWT has demonstrated significant anti-inflammatory effects in various animal and clinical settings, and its mechanism is increasingly recognized to involve coordinated modulation of multiple signaling pathways rather than a single-target effect (9, 24–26, 32). Nitric oxide (NO) in animals is a substance that has both pro-inflammatory and anti-inflammatory effects. When the concentration of NO in the body is within the physiological range, it can exert an anti-inflammatory effect. Current evidence indicates that ESWT not only upregulates endothelial nitric oxide synthase (eNOS) activity and NO production to suppress NF-κB activation and downstream inflammatory gene expression (24) but also regulates several other signaling cascades, including the PI3K/Akt/FOXO1 (9), integrin–FAK–p38 MAPK (25), and toll-like receptor 3 (TLR3) pathways (32). Feng B found that ESWT can inhibit inflammation by regulating the PI3 K/AKT/FOXO1 signaling pathway. Specifically, ESWT significantly reduced TNF-α, IL-6, IL-1β, COX-2 and SP in the tissues of Chronic Prostatitis/Chronic Pelvic Pain Syndrome rats, while inhibiting the expression of p-AKT and p-FOXO1, and the expression of catalase increased (9). In 2023, Ge et al. (25) found that ESWT could upregulate the phosphorylation of FAK at the Tyr397 site and downregulate the phosphorylation of p38MAPK at the Thr180 and Tyr182 residues. The researchers also further used integrin inhibitors to intervene and confirmed that ESWT inhibited the secretion of IL-1β through the integrin-FAK-p38MAPK pathway to achieve the purpose of limiting the development of inflammation. ESW also regulates IL-6 and IL-10 by activating the TLR3 pathway to control the development of inflammation in the body. After ESWT (250 pulses, 0.08 mJ/mm^2^ and 3 Hz) acted on HUVEC cells, Cyclophilin B (CYP B) expression was upregulated, allowing cells to take up more nucleic acids. CYP B binds to the TLR3 receptor to produce more TLR3 mRNA to activate TLR3. After TLR3 activation, IL-6 further mediates early proinflammatory responses and acts as a chemokine to attract monocytes to the inflammatory site to initiate inflammatory responses. Vascular cell adhesion molecules were not upregulated, but IL-6 levels increased, indicating that the inflammatory response did not continue to intensify after ESWT treatment. In addition, ESWT treatment activated TLR3 and significantly increased the anti-inflammatory cytokine IL-10, further inhibiting the development of inflammation (32). All in all The activation or inhibition of these pathways in different models has been associated with reduced levels of proinflammatory cytokines [such as TNF-α (9, 24), IL-6 (9, 32), IL-1β (9, 25), COX-2 (9, 24), and substance P (SP) (9)] and increased catalase activity (9) with the upregulation of the anti-inflammatory cytokine IL-10 (32), thereby limiting the progression of inflammation.

In addition to signaling pathway modulation, ESWT also appears to influence immune cell phenotypes. Low-energy ESWT, *in vitro* experiments, can suppress the expression of inflammatory genes and chemokines in M1 macrophages while simultaneously promoting the expression of M2 macrophage markers and IL-10, thus enhancing anti-inflammatory effects at the immunoregulatory level (33). In burn models, ESWT markedly downregulates the expression of multiple chemokines (CCL2, CCL7, CXCL2, CXCL5, and CXCL28), acute-phase cytokines (IL-1β, IL-6, and IFNβ), and matrix metalloproteinases (MMP-3, MMP-9, and MMP-13), thereby attenuating wound inflammation and facilitating repair (34).

Taken together, these findings suggest that ESWT exerts anti-inflammatory effects through a dual mechanism of signaling pathway modulation plus immune-cell phenotype conversion. This multifaceted mode of action provides a theoretical basis for its veterinary and clinical applications, although the key molecular mediators and their interactions require further elucidation.

### 2.3 Self-repair mechanism

ESWT acts synergistically through promoting cell proliferation and matrix synthesis (35, 36), angiogenesis (28, 37, 38), and gene regulation (39, 40), thereby increasing tissue responsiveness to injury and enabling self-repair. Its reparative effects have been demonstrated in bone (29), soft tissue (30), and cartilage (21).

Specifically, at the cellular level, ESWT markedly stimulates proliferation and matrix synthesis (35, 36). Mechanistically, ESWT upregulates proliferating cell nuclear antigen (PCNA), transforming growth factor-β1 (TGF-β1), insulin-like growth factor-I (IGF-I), and various cyclins while inducing ATP release, which collectively enhances cellular activity and matrix deposition (35). In addition to enhancing the early expression of hyaluronic acid, ESWT promotes regeneration and bone healing by increasing the concentration of sulfated glycosaminoglycans and prolonging their anabolic phase, thereby directly modulating matrix metabolism to support tissue regeneration and bone repair (36).

With respect to angiogenesis, ESWT regulates key molecules such as vascular endothelial growth factor (VEGF) (28, 37), eNOS (28, 37), and VEGF receptor-2 (38), thereby improving local perfusion and promoting neovascularization. Notably, wound healing is markedly delayed in eNOS-deficient mice following ESWT, underscoring the pivotal role of eNOS in angiogenic processes (37).

In addition to these effects, ESWT exerts multilevel reparative effects through broad gene regulatory mechanisms (39, 40). In animal models of osteoarthritis, for example, it modulates numerous genes in bone and cartilage involved in cartilage development, inflammatory responses, angiogenesis, and cell proliferation (39). Concurrently, ESWT upregulates myogenic regulatory genes such as MyoD and myosin, thereby facilitating myofiber regeneration and muscle repair (40). Currently, the mechanisms by which ESWT promotes the body’s self-repair remain incompletely understood. However, the synergistic interplay of multidimensional repair mechanisms forms the biological basis for realizing ESWT’s self-repair potential and provides theoretical support for its application in the treatment of musculoskeletal injuries.

## 3 Clinical application and mechanism of ESWT in equine musculoskeletal diseases

A survey conducted by the American Association of Equine Practitioners (AAEP) among equine veterinarians in U. S. clinical practice reported that 37% used ESWT at least once a week to treat equine musculoskeletal injuries (17). Veterinarians can choose different ESW devices, action sites, treatment times, and ESW-related parameters to develop personalized treatment plans to maximize the treatment effect (Table 1).

**Table 1:** Summary of studies and their results.

Disease, lameness scores	Clinical sample information	ESWT treatment plan	Probe focal length	Treatment times and interval time	Therapeutic effect	Researcher (Time)
Proximal suspensory desmitis	49 Horses (Quarter horses 47, Arabians 2; mean 6 years, range 2–22 years)	Focused ESWT (E6), 800 pulses	20 mm probe	13 horses received only the first treatment, 13 horses received two treatments, and 21 horses received three treatments	ESWT therapy is superior to PRP	Giunta et al. (2019) (16)
Chronic proximal suspensory desmitis	56 Horses (Warmblood 44, Arabian 8, Thoroughbred 3, Pony 1; 2–22 years, mean 10.3 years)	Focused ESWT (Electrohydraulic), 0.15 mJ/mm^2^, 2000 pulses at the origin of the suspensory ligament	R35 probe	Treatment was given once every 3 weeks for a total of three times.	21 (61.8%) forelimb cases and 22 (40.9%) hindlimb cases returned to full work six months after diagnosis. 19 (55.9%) forelimb cases and 4 (18.2%) hindlimb cases were still able to work fully one year after ESWT treatment.	Lischer et al. (2006) (2)
Proximal suspensory desmitis	65 Horses (2–17 years, 78% aged 6–12 years)	Radial pressure wave therapy, 1,000 pulses each on the medial and lateral aspects of the limb (directed at the origin of the suspensory ligament) (Swiss Dolorclast Vet machine)	——	Horses were treated 3 times at 2-week intervals	Forty-one percent of horses with hindlimb lameness and 53% with forelimb lameness were nonlame and returned to full work 6 months after diagnosis.	Crowe et al. (2004) (3)
Collagenase induced hind limb suspensory ligament desmitis.	10 Horses	Focused ESWT, 0.15 mJ/mm^2^, 500 pulses each on lateral (5 mm probe)、medial (5 mm probe)、plantar (35 mm probe) aspects of the mapped suspensory ligament	5 mm and35 mm probe	Treatment was given once every 3 weeks for a total of three times.	The number of collagen fibers and extracellular matrix components in the diseased ligaments increased after ESWT.	Caminoto et al. (2005) (42)
Collagenase-induced equine forelimb suspensory ligament desmitis	4 Horses (Quarter horses 4; mean 7.25 years)	Focused ESWT, 0.14 mJ/mm^2^, 500 pulses each on palmar-lateral (5-mm probe)、palmar-medial (5-mm probe)、palmar (35-mm probe) aspects; Affected area includes PSD centered on the lesion extending outwards for ten centimeters.	5 mm and 35-mm probe	Treatment was given once every 3 weeks for a total of three times.	Ultrasound and histology showed recovery to normal function	McClure et al. (2004) (43)
OA of the pastern joint Grade 3/5 lameness (AAEP)	1 Horse (Holsteiner/Selle Francais; 10 years old)	Focused ESWT, energy level 9, 1,000 pulses with a 35 mm focal depth probe and 1,000 pulses with a 5 mm focal depth probe; action site: pastern joint. (Equitron; High Medical Technologies USA [HMT], Marietta, Georgia)	5 mm and 35 mm probe	A total of three ESWT were performed	The decreased lameness scores lasted approximately 2 years.	Revenaugh (2005) (4)
Hock arthritis of the tarsometatarsal and distal intertarsal joints	1 Horse (Paint Horses; 12 years old)	Focused ESWT, energy level 9; regionally applied over the affected distal tarsal bones and joints (Equitron; High Medical Technologies USA [HMT], Marietta, Georgia)	20 mm probe	The interval between the two treatments is about 4 weeks	ESWT enhanced the horse’s performance with minimal convalescent time	Revenaugh (2005) (4)
Hock arthritis of the tarsometatarsal and distal intertarsal joints Grade 1/5 lameness (AAEP)	1 Horse (Quarter Horse; 10 years old)	Focused ESWT, energy level 9, 1,500 pulses applied to the lesion site (Equitron; High Medical Technologies USA [HMT], Marietta, Georgia)	20 mm probe	Treatment was given once every 3 weeks for a total of three times.	The lameness was significantly improved.	Revenaugh (2005) (4)
Carpal arthritis and synovitis Grade 2/5 lameness (AAEP)	1 Horse (Quarter Horse; 10 years old)	Focused ESWT, energy level 9, 700 pulses with 5 mm and 35 mm focal length probes, respectively, on the dorsal and medial aspects of each carpus (Equitron; High Medical Technologies USA [HMT], Marietta, Georgia)	5 mm and 35 mm probe	Treatment once a month for a total of three times	Lameness and swelling reduced	Revenaugh (2005) (4)
Capsulitis and softening of the dorsal aspect of the proximal first phalanx Grade 3/5 lameness (AAEP)	1 Horse (Arabian horse; 10 years old)	Focused ESWT, energy level 4 (of 6), applied to the lesion site (VersaTron High Medical Technologies USA [HMT], Marietta, Georgia).	5 mm probe	Treatment once a week for a total of three times.	The lameness was significantly improved.	Revenaugh (2005) (4)
Bone spavin Lameness grades ranging from 1 to 3(AAEP)	74 Horses (Quarterhorses 59, Paint Horses 13, Appaloosa, Thoroughbred; 18 months–22 years, median 4 years old)	Focused ESWT (electrohydraulic), 0.89 mJ/mm^2^, 2000 pulses applied to the lesion site.	——	——	There was a decrease in the lameness of 80% (59/74) of the horses treated.	Mccarroll and Mcclure (2002) (19)
Arthroscopic-induced Osteoarthritis in Middle Carpal Joints mean ± SEM lameness score, 2.20 ± 0.11	24 Horses (2–3 years old)	Focused ESWT (electrohydraulic): 1st treatment with 0.14 mJ/mm^2^ (800 pulses, over carpal joint capsule) + 400 pulses over osteoarthritis fragment area; 2nd treatment with 0.15 mJ/mm^2^ (600 pulses over carpal joint capsule) + 300 pulses over osteoarthritis fragment area.	——	Treatment was given once every 2 weeks for a total of two times.	Significant improvement in lameness but no significant improvement in horse synovial fluid, synovium or cartilage.	Frisbie et al. (2009) (45)
Navicular Syndrome The mean + SEM lameness grade:1.9 + 0.18 (AAEP)	27 Horses (Quarter horses 11, Thoroughbreds 5, Paint Horses 4, warmbloods 4, Appaloosas 2, American Saddlebred 1; median 12 years old, range 3–20 years old)	Focused ESWT (electrohydraulic), 0.89 mJ/mm^2^, 1,000 pulses each through the frog and the heel	——	Received once ESWT	Significantly improves lameness symptoms in horses	Mcclure et al. (2024) (48)
Navicular Syndrome Mair score:1–5 Turner–Tucker score:9–23	42 Horses (Warmbloods of different breeds 35, Quartes 5, German riding pony 1, Thoroughbred crossbred horse 1; mean 12 years, range 5–19 years)	Focused ESWT (electrohydraulic), 0.15 mJ/mm^2^; Group A: 1200 pulses through the sole of the hoof; Group B: 1200 pulses through the bulb region	——	Received once ESWT	80% treated through the bulb region no longer showed lameness, and 47.4% treated through the sole of the hoof no longer showed lameness.	Blum et al. (2005) (5)
Navicular Syndrome	9 Horses (Quarter horses 8, Thoroughbred 1; 5–12 years old)	Radial pressure wave therapy, 1,500 pulses between the heel bulbs and 1,500 pulses over the middle third of the frog (Swiss Dolor Clast Vet radial machine, Electro Medical Systems, Dallas, TX)	——	Received once ESWT	ESWT did not improve lameness	Brown et al. (2005) (49)
Back pain syndrome	12 Horses (Thoroughbred 5, Hanoverian 2, Westphalian, Trakehner, Quarter Horse, Thoroughbred/Quarter Horse, Irish Sport Horse; median 14.5 years, range 7–19 years)	0.13 mJ/mm^2^, 750 pulses per thoracolumbar segment (T12-L5)	——	Treatment was given once every 2 weeks for a total of three times.	The mechanical pain threshold detected each time was higher than that before the first ESWT treatment.	Trager et al. (2020) (7)
Superficial digital flexor tendinitis (AAEP)	6 Horses (mean 7.2 years, range 3–18 years)	Focused ESWT, 0.14 mJ/mm^2^, 1,500 pulses on the injured area (8 cm to 20 cm distal to the accessory carpal bone)	——	Treatment was given once every 3 weeks for a total of three times.	ESW did not change the clinical or ultrasonic manifestations of superficial digital flexor tendinitis but increased the formation of new blood vessels.	Kersh et al. (2006) (51)

ESWT, Extracorporeal Shock Wave Therapy; PRP, Platelet-Rich Plasma; AAEP, American Association of Equine Practitioners; PSD, Proximal suspensory desmitis.

**Figure 1:**
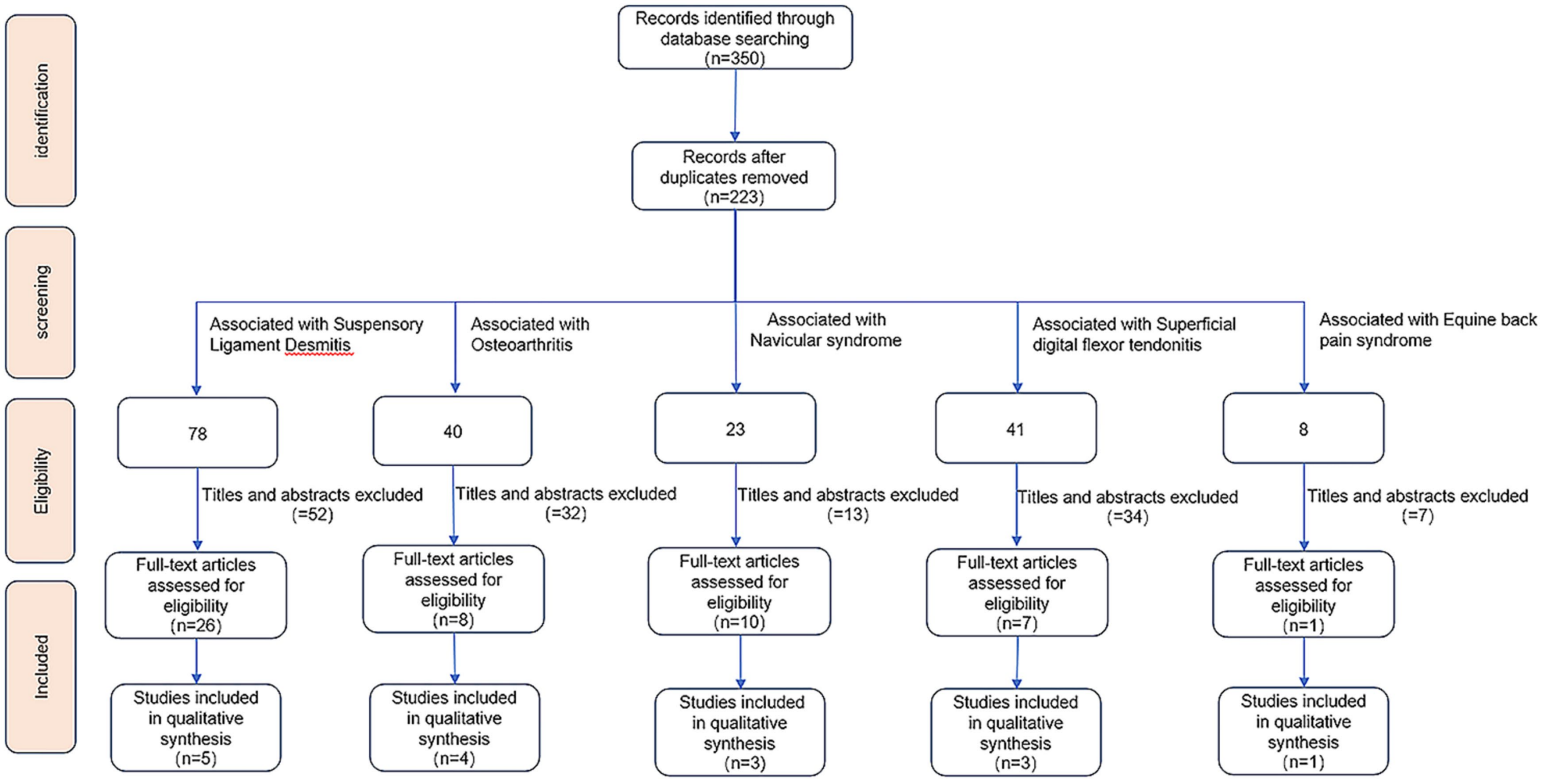
Literature retrieval flowchart depicting the selection and exclusion of studies.

The search was conducted in the PubMed, Web of Science, and CABI Digital Library databases. The search time ranged from the inception of the database to March 2025. The specific method is shown in Figure 1. The search terms are shown in Supplementary Table S1.

The search was conducted independently by two authors, Zhongsheng Qiu and Jiaqi Wang. First, duplicate articles were removed. Then, potentially relevant articles were retained by screening titles and abstracts. Finally, relevant articles were identified through full-text analysis, and the two authors discussed differences to reach a consensus. Potentially relevant articles were also retrieved from the reference lists of the included articles.

The inclusion criteria were as follows: (1) the main disease was osteoarthritis, suspensory ligament desmitis, tendinitis, navicular syndrome or back pain syndrome; and (2) the affected horses received ESWT treatment.

The exclusion criteria were as follows: (1) letters, editorials, comments, or reviews; and (2) nonequine studies.

### 3.1 Suspensory ligament desmitis in horses

#### 3.1.1 Clinical practice of ESWT for equine suspensory ligament desmitis

Suspensory ligament injuries are among the most common causes of lameness in horses, with a particularly high incidence in racehorses. Statistics show that approximately 16.1 suspensory ligament injuries occur per 1,000 race starts (41). Current treatment modalities for suspensory ligament injuries include ESWT, and platelet-rich plasma (PRP) therapy. A two-center, randomized, prospective clinical trial compared the efficacy of ESWT and PRP in the treatment of Proximal suspension desmitis (PSD) in horses with Western performance. Veterinarians applied a focused shock wave generator (VersaTron99) with a 20-mm probe delivering 800 pulses at the E6 energy level. Three treatment sessions at one-week intervals were recommended; however, the actual number of sessions administered ranged from one to three depending on the owners’ preferences. At 4 days post-treatment, the improvement in lameness scores was greater in the ESWT group (47.23 ± 5.61) than in the PRP group (32.48 ± 5.26). At 6 months post-treatment, both groups showed significant improvement in lameness scores, and no significant difference in working ability was observed. At 1 year, the possibility of returning to work for PSD in the ESWT group was 3.8-fold greater than that in the PRP group (*p* = 0.05) (16). While this study suggests that PRP may be more advantageous than ESWT for long-term management, it did not clarify the specific concentrations of components in PRP. Different PRP products vary significantly in their composition; furthermore, the injection dosage depends on the veterinarian’s clinical judgment. Therefore, the heterogeneity of PRP and the influence of treatment dosage on efficacy cannot be excluded. And, the small number of horses with severe PSD in this study could easily lead to bias in the results. Future comparative studies of ESWT and PRP should include comparisons of treatment effects on horses with different severities (especially severe cases) of PSD, to better determine the optimal long-term or short-term treatment plans for horses with different degrees of PSD.

Currently, two studies have reported that ESWT produces differential therapeutic outcomes in chronic PSD of the forelimbs and hindlimbs, with overall superior efficacy observed in forelimb cases (2, 3). This difference has been attributed to the greater susceptibility of the hindlimb suspensory ligament to compression from surrounding tissues, potentially leading to a compartment syndrome–like condition associated with chronic pain (2). However, discrepancies exist between these studies, as one employed focused ESWT while the other used radial pressure wave therapy. Overall, the efficacy of radial pressure wave therapy was lower than that of focused ESWT (3). This phenomenon is likely due to the differences in shock wave modalities: focused shock waves have a deeper action site and minimal energy loss, whereas radial shock waves undergo substantial attenuation during transmission, with only a small fraction of the energy reaching the lesion site.

#### 3.1.2 Mechanism of action

Two studies using collagenase-induced PSD models in horses demonstrated that repeated ESWT promotes ligament repair (42, 43). Treatments were administered once every 3 weeks for a total of three sessions, with an energy flux density of 0.14–0.15 mJ/mm^2^. In each session, 500–1,500 pulses were applied to key regions of the proximal suspensory ligament, including the medial, lateral, and palmar/plantar aspects (42, 43). Compared with controls, ESWT significantly reduced the lesion area (42, 43), and histological analysis revealed increased deposition of fine collagen fibrils (42), mitochondria (42), and proteoglycans (43), indicating enhanced tissue repair. The reparative effects of ESWT likely involve multiple mechanisms. It upregulates TGF-β1 expression at the lesion site, stimulating the synthesis of fine collagen fibrils (42). ESWT also promotes mitochondrial biogenesis and enhances cellular metabolic activity (42) while augmenting the local secretion of collagen, extracellular matrix components, and growth factors (42). In addition, ESWT appears to induce fibroblast aggregation at the lesion site and stimulate proteoglycan secretion, coordinating the remodeling of ligament collagen fibers and accelerating repair and regeneration (43).

#### 3.1.3 Recommendations and precautions

For the treatment of PSD in horses, controlling local inflammatory reactions through controlled exercise, hydrotherapy, and systemic or local application of nonsteroidal anti-inflammatory drugs is recommended. ESWT or PRP therapy is selected to repair damaged parts according to the severity of ligament injury. ESWT is used for horses with mild and moderate lameness, whereas PRP therapy is used for horses with severe lameness. The reference treatment plan for ESWT in equine PSD is to use a focused shock wave generator to generate ESW with an energy flux density of 0.14–0.15 mJ/mm^2^, which is applied to the lesion under the guidance of ultrasound, with 1,500–2000 pulses given per treatment (42, 43). Horses with acute PSD should be treated with ESWT every 1–2 weeks, whereas horses with chronic PSD should be treated with ESWT every 2–3 weeks. A total of three ESWT treatments can be performed, and the number of ESWT treatments can be increased or decreased depending on the treatment effect. Prior to a horse returning to work, ultrasonographic examination of the suspensory ligament in the affected limb is required to assess whether its tissue architecture exhibits a trend of stable improvement. Notably, ultrasonographic examination cannot fully confirm that the suspensory ligament has returned to a healthy state; a comprehensive evaluation in conjunction with clinical manifestations is necessary. This is to prevent re-injury of the suspensory ligament and the occurrence of fascial compartment-like syndrome resulting from premature return to work. In veterinary clinical treatment, appropriate treatment plans should be formulated for different cases on the basis of individual differences, such as whether PRP or ESWT should be used, ESWT parameters, the number of ESWT treatments, and the rest time.

### 3.2 Osteoarthritis (OA)

#### 3.2.1 Clinical practice of ESWT in equine OA

Equine OA is a common condition that impairs equine locomotor function. Clinical veterinarians often use systemic or local injections of nonsteroidal anti-inflammatory drugs to relieve joint pain and inhibit the development of local inflammation in affected horses.

ESWT has demonstrated promising efficacy in the management of equine OA. High-energy ESWT can significantly reduce lameness scores and restore locomotor function, even in patient’s refractory to conventional treatments (4, 19). In a study conducted by Revenaugh MS, five horses with advanced OA that had failed to respond to other treatments were treated with ESWT. Only one horse experienced recurrence 2 years later due to trauma, and repeat ESWT was ineffective (4). In a separate retrospective study involving 74 horses with spavin (hock osteoarthritis), a single ESWT session was applied to the lesion (energy flux density 0.89 mJ/mm^2^, 2000 pulses), followed by a short period of stall rest and gradual return to exercise through walking and groundwork. At 12 weeks post-treatment, 38% of the horses improved by one lameness grade, 42% improved by two grades, and 18% achieved complete recovery; no recurrences were observed during the 90-day follow-up (19). Radiographic evaluation revealed no significant changes in joint morphology or accelerated fusion. ESWT promotes bone formation by modulating the differentiation of osteoblasts and osteoclasts (44), which may indicate that ESWT enhances subchondral bone strength, preserves joint morphology, and mitigates cartilage damage.

#### 3.2.2 Mechanism of action

Polysulfated glycosaminoglycan (PSGAG) is a commonly used therapeutic agent for equine OA and has been clinically validated to alleviate lameness and joint symptoms to some extent. Frisbie et al. conducted a randomized controlled trial comparing the efficacy of ESWT, PSGAG, and placebo in horses with OA induced by arthroscopic surgery. ESWT produced a significant reduction in lameness scores within 2 weeks of treatment, which was maintained throughout the 70-day study period, whereas lameness scores in the PSGAG group did not differ significantly from those of the placebo group throughout the trial. Although the carpal flexion response tended to be better in the ESWT group than in the other two groups, this difference did not reach statistical significance. No significant differences were detected among the three groups in synovial fluid analyses or in the gross and histologic evaluation of the cartilage or synovium, except for a slight decrease in total protein in the synovial fluid of the ESWT and PSGAG groups compared with the placebo group (45). Subsequent studies further indicated that ESWT may promote subchondral bone remodeling, enhance bone strength, and relieve pain by increasing the serum osteocalcin and C-terminal telopeptide of type I collagen. ESWT has also been shown to increase proteoglycan expression in synovial fluid, thereby helping to prevent further cartilage damage (46). ESWT was superior to PSGAG in the treatment of horses with experimentally induced OA. However, this study only lasted 70 days, and it did not find that ESWT or PSGAG exerted a cartilage-repairing effect. The study used an osteoarthritis model induced by cartilage detachment, and this type of intense mechanical insult may have made it difficult for PSGAG to exert structural repair effects, thus potentially leading to an underestimation of PSGAG’s efficacy. It is recommended that future related studies extend the follow-up period to observe treatment effects, to clarify whether ESWT and PSGAG induce structural improvements in bone or cartilage, and to conduct a comparative evaluation of ESWT and PSGAG in horses with naturally occurring OA.

#### 3.2.3 Analysis and suggestions

From the perspective of clinical diagnosis and pathology, ESWT is superior to PSGAG in the treatment of equine OA. However, the effect of ESWT in the treatment of equine OA in the Frisbie DD study is different from that reported by McCarroll GD and Revenaugh MS. This difference may be attributed to the relatively low energy flux density (0.14–0.15 mJ/mm^2^) used by the Frisbie DD team during the treatment process, which affects the therapeutic effect of ESWT. This may also be due to the difference in treatment effects caused by different types of OA horses. The horses treated by McCarroll GD and Revenaugh MS were all advanced OA horses after failure of other treatments, whereas the Frisbie DD team established the equine OA model through surgery. In addition, horses with OA require timely rest. In the studies by McCarroll GD and Revenaugh MS, the horses received ESWT followed by exercise restriction. In contrast, the horses in Frisbie DD’s study began simulating intense exercise consistent with racehorse training from the second day post-treatment until the end of the study. This may also be one of the factors contributing to the differences in treatment outcomes.

According to published studies, ESWT for equine OA is recommended to be performed via a high-energy focused shock wave generator that delivers 1,000–2,000 pulses to the affected area. The energy flux density is typically set at 0.14–0.89 mJ/mm^2^, with higher energy levels indicated for chronic cases of OA (19, 45). During treatment, an individualized protocol should be developed to account for interhorse variability and thereby maximize the therapeutic efficacy of ESWT.

### 3.3 Navicular syndrome

#### 3.3.1 Clinical practice of ESWT in navicular syndrome patients

Navicular syndrome is one of the most common hoof diseases in horses and severely affects their athletic performance. It is frequently associated with factors such as excessive exercise, inappropriate hoof trimming, and overweight, as well as other biomechanical and conformational abnormalities (47). Currently, conservative treatments for this disease mainly include systemic nonsteroidal anti-inflammatory drug administration, ESWT, hoof trimming and other interventions.

ESWT appears to be an effective intervention for refractory equine navicular syndrome (5, 48). In one investigation, horses received a single ESWT session at an energy flux density of 0.89 mJ/mm^2^, during which 1,000 pulses were delivered to both the frog and the hoof. Following treatment, the horses were confined to stall rest for 1 week and then subjected to 5 weeks of hand walking and arena acclimatization before they returned to full activity. Lameness grades improved in 81% (22/27) of horses in nonblinded evaluations, whereas blinded assessments documented improvement in 56% (9/16) of cases (48).

Interestingly, another study suggested that ESWT efficacy may depend on the anatomical site of application. Blum N. applied shockwaves either to the heel bulb region or to the sole of the hoof. The proportion of horses that became sound was 80% when treated exclusively at the heel bulb and 47.4% when treated solely at the sole (5). This discrepancy may be attributable to differences in tissue characteristics at the application sites: the sole contains a higher keratin content than the heel bulb region does, potentially attenuating shockwave energy during transmission. Nevertheless, these findings should not be interpreted as justification for uniformly applying ESWT only to heel bulb in clinical practice, as this approach would limit stimulation of the navicular bone. A more appropriate strategy may be to increase either the number of pulses or the energy level delivered to the sole to ensure an adequate therapeutic effect.

#### 3.3.2 Discussion and suggestions

Most clinical reports on ESWT for navicular syndrome suggest that ESWT can produce good therapeutic effects, but in a prospective study by Brown KE, ESWT did not improve lameness in horses. Brown KE chose to use a radial machine to generate ESW (pressure: 4 bar, frequency: 10 Hz). The 1500 pulses were applied between the heel bulbs with the foot on the ground, and the other 1,500 pulses were applied over the middle third of the frog with the limb elevated (49). The reason for the difference in the treatment results may be the use of a radial ESWT generator. The radial ESW is prone to energy loss during transmission, resulting in insufficient stimulation of the navicular, leading to poor therapeutic effects of ESWT. According to the aforementioned research report on ESWT treatment of equine navicular syndrome, using focused ESW to comprehensively target the lesion site through heel bulbs and sole is an effective treatment method (5, 48, 49).

### 3.4 Superficial digital flexor tendinitis

#### 3.4.1 Clinical practice of ESWT for equine superficial digital flexor tendinitis

Superficial digital flexor tendinitis (SDFT) is one of the most common tendon diseases in high-performance horses. This degenerative disease is caused by long-term overstretching of the tendon and accounts for approximately 46% of all limb injuries in racehorses (50). ESWT has shown great potential in the field of equine SDFT treatment because of its unique biological effects. A survey showed that 76.8% (96/125) of respondents (predominantly equine clinical veterinarians in the United States) utilize ESWT for the treatment of equine tendon injuries, and 71.4% (95/133) of respondents consider that ESWT exerts a positive effect on the recovery of equine tendon injuries. Furthermore, 73.6% (92/125) of the respondents used focused shock wave generators (17). In a retrospective study, ESWT (0.15 mJ/mm^2^, 1,000–2000 pulses) was found to significantly reduce the degree of lameness in horses with SDFTs and PSD. In addition, no significant differences in short-term or long-term outcomes were found between the different numbers of ESWT treatments (6). However, the authors believe that this conclusion is not rigorous. In their study, the degree of lameness and duration of lameness in horses in group one (<3 ESWT) were lower than those in group two (≥3 ESWT). In addition, the number of cases included in the two treatment groups (group one: 8 horses, group two: 14 horses) was quite different. The results of the small sample size treatment group are prone to bias, thus affecting the overall conclusion of the study.

#### 3.4.2 Mechanism of action

The mechanism of ESWT in treating equine SDFT is still unclear. However, some studies have shown that ESWT can significantly increase angiogenesis at the SDFT injury site induced by collagenase and increase the level of extracellular matrix secreted by tenocytes (51). The newly formed blood vessels provide more nutrients and oxygen to the injury site while promoting the removal of local metabolic waste, which helps accelerate tendon healing and may relieve pain associated with the injury. The extracellular matrix is essential for maintaining the structure and function of the tendon, and its increased secretion helps improve the quality of tendon healing. In addition, ESWT may promote collagen degradation, remodeling, and new collagen synthesis by destroying the collagen matrix and upregulating the expression of the COLI and MMP14 genes, thereby repairing the tendon (52).

#### 3.4.3 Recommendations

On the basis of literature reports, a focused shock wave generator should be used during ESWT treatment of equine SDFT, the energy flow density should be set to 0.13–0.15 mJ/mm^2^, and 1,000–2,000 pulses should be applied to the affected area (6, 51). ESWT should be performed three or more times, with an interval of one to 2 weeks between each time. Treatment parameters and the number of treatments need to be adjusted appropriately according to the individual condition of the horse and the disease.

### 3.5 Equine back pain syndrome

#### 3.5.1 Clinical practice of ESWT for equine back pain syndrome

Equine back pain syndrome, or equine back disorder, is a common condition in horses that can be caused by a number of factors (impinging dorsal spinous processes, desmopathy of the supraspinous ligament, osteoarthritis of the articular processes, and soft tissue injuries) (53). One survey reported that back pain was second only to lameness in terms of incidence among endurance horses, with 35.2% (67/190) of horses experiencing back pain at some point in their careers (54).

Trager LR conducted a nonrandomized controlled trial to explore the possible mechanism of ESWT in treating equine back pain. During the trial, the affected horses received three ESWTs (0.13 mJ/mm^2^, 1,500 pulses, and ESWT every 2 weeks). The probe formed a 45° angle with the spine, and ESW was applied to both sides of T12--L5. The results showed that ESWT achieved analgesia by increasing the mechanical nociceptive threshold of the horse, but the cross-sectional area of the multifidus muscle of the affected horse did not significantly change (7).

#### 3.5.2 Recommendations

The occurrence of equine back pain is caused by a variety of factors, so it is necessary to develop a corresponding personalized treatment plan on the basis of the different primary diseases of affected horses. Allen AK recommends the use of a high-energy focused shockwave machine to deliver 1,000–2000 pulses to the affected area. For back pain caused by spinous process impingement, a 35 mm probe should be placed just above and to the side of the thoracic and lumbar spinous processes. For back pain caused by dorsal articular process OA, an 80 mm probe is recommended to be placed to the left and right of the affected area (18). In terms of prognosis, it is recommended that patients with equine back pain syndrome be treated with ESWT once or twice a year as part of the horse’s annual maintenance program. This helps maintain the overall health of the back and spine, and by maintaining the back, overall freedom of movement can be improved, thereby improving gait, overall health, and quality of life.

## 4 ESWT combined with regenerative medicine therapy

ESWT is a safe and effective way to treat equine musculoskeletal injuries. However, owing to individual differences in horses, ESWT does not produce ideal therapeutic effects for all horses. Combining ESWT with other therapies has become a feasible strategy to improve treatment efficacy. PRP therapy is a regenerative medicine treatment for equine soft tissue injuries. Recent studies have shown that ESWT acting on PRP can significantly increase the release of transforming growth factor-β1 and platelet-derived growth factor ββ (55). This finding suggests that ESWT combined with PRP therapy can be used for horses that do not respond well to ESWT or PRP monotherapy in clinical practice. Combined treatment may significantly improve the repair efficiency of damaged tissues through the dual mechanisms of synergistically promoting local angiogenesis and inhibiting inflammatory responses. In addition, the increase in the release of growth factors can also accelerate tissue repair. However, the specific mechanism by which ESWT promotes the release of growth factors from platelet is still unclear and needs further exploration by researchers.

With the development of regenerative medicine, stem cell therapy has provided a new option for the treatment of equine musculoskeletal injuries. Currently, commercially available stem cell products are being used to treat musculoskeletal system injuries in horses. Stem cell therapy can improve the healing quality of damaged tissues and reduce the possibility of recurrence. Salcedo-Jimenez R reported that Horse Cord Blood Mesenchymal Stromal Cells had increased metabolic, adipogenic, and osteogenic activities after being treated with ESW, but their immunosuppressive properties did not change (56). In a study by Colbath AC, no sustained osteogenic effect was observed in horse bone marrow-derived mesenchymal stem cells treated with ESW (57). Neither researcher have reported that ESWT has an adverse effect on stem cell proliferation. The combination of ESWT and stem cell therapy is expected to promote tissue regeneration and repair through multiple synergistic mechanisms and improve clinical efficacy.

## 5 Limitations

This review aims to comprehensively summarize the research progress of ESWT in equine musculoskeletal diseases, but some limitations remain. Some studies are characterized by small sample sizes, short follow-up periods, inconsistent inclusion criteria, lack of blinded assessments, and differences in rehabilitation protocols, these factors may introduce bias into efficacy assessments. Furthermore, some studies use different disease outcome assessment indicators (such as serum biomarkers and histological details); however, core assessment indicators remain consistent, including lameness scores and tissue repair effects, which can mitigate bias. In addition, some studies do not provide full details of ESWT parameters, hindering comparisons with other studies. Moreover, current studies often rely on subjective experience to set parameters, leading to inconsistencies across studies, a feature inherent to the current stage of development in this field. When synthesizing multiple studies, this review attempted to minimize the impact of such inconsistencies by comparing different parameter backgrounds. Overall, these limitations highlight the need for further improvements in future research but do not undermine the core conclusions of this review regarding the biological effects and clinical application value of ESWT.

## 6 Conclusion

ESWT has become an important means of treating equine musculoskeletal injuries because of its multiple biological effects, such as analgesia, anti-inflammatory effects, promotion of angiogenesis and tissue repair. However, we need to be vigilant about the potential side effects (decreased cell viability (44), mechanical changes (52), collagen matrix disorder (58)) of ESWT, which may cause local adverse reactions and damage normal tissues. Therefore, during the treatment process, it is essential to accurately localize the target site; optimize treatment parameters—including the energy flux density, number of pulses, treatment interval, and number of sessions. Additionally, appropriate post-ESWT rehabilitation protocols—such as controlled walking, hydrotherapy, and balance training—should be implemented to promote physiological remodeling, reduce tissue adhesion, restore locomotor function, and thereby maximize therapeutic efficacy while minimizing potential risks. Future research should delve into its mechanism of action and long-term effects, and explore combinations with other therapies to enhance efficacy and reduce adverse effects. This will provide reference protocols for ESWT in the management of equine musculoskeletal injuries.
