# Supplementary material for: Extracorporeal shock wave therapy for equine musculoskeletal disorders: from biological mechanisms to clinical applications
Source: Front Vet Sci. 2025 Dec 19;12:1719123. doi: 10.3389/fvets.2025.1719123 (PMC12757233; doi:10.3389/fvets.2025.1719123)
Supplement: Supplementary file 1 [file Table_1.docx]

Table S1: Summary Table of Search Terms for the Systematic Review

| Suspensory ligament desmitis | ((Ligament) OR (Interosseal Ligament) OR (Interosseal Ligaments) OR (Ligament, Interosseal) OR (Interosseous Ligament) OR (Interosseous Ligaments) OR (Ligament, Interosseous) OR ("Ligaments"[Mesh])) AND ((Horse) OR (Equus caballus) OR (Horse, Domestic) OR (Domestic Horse) OR (Domestic Horses) OR (Horses, Domestic) OR (Equus przewalskii) OR ("Horses"[Mesh])) AND (("Extracorporeal Shockwave Therapy"[Mesh]) OR (Extracorporeal Shockwave Therapies) OR (Shockwave Therapies, Extracorporeal) OR (Shockwave Therapy, Extracorporeal) OR (Therapy, Extracorporeal Shockwave) OR (Extracorporeal Shock Wave Therapy) OR (Shock Wave Therapy) OR (Shock Wave Therapies) OR (Therapy, Shock Wave) OR (Extracorporeal High-Intensity Focused Ultrasound Therapy) OR (Extracorporeal High Intensity Focused Ultrasound Therapy) OR (High-Intensity Focused Ultrasound Therapy) OR (High Intensity Focused Ultrasound Therapy) OR (HIFU Therapy) OR (HIFU Therapies) OR (Therapy, HIFU)) |
| --- | --- |
| Osteoarthritis | ((Osteoarthritides) OR (Arthritis, Degenerative) OR (Arthritides, Degenerative) OR (Degenerative Arthritides) OR (Degenerative Arthritis) OR (Osteoarthrosis) OR (Osteoarthroses) OR (Osteoarthrosis Deformans) OR (Arthrosis) OR (Arthroses) OR ("Osteoarthritis"[Mesh]) OR (spavin)) AND ((Horse) OR (Equus caballus) OR (Horse, Domestic) OR (Domestic Horse) OR (Domestic Horses) OR (Horses, Domestic) OR (Equus przewalskii) OR ("Horses"[Mesh])) AND (("Extracorporeal Shockwave Therapy"[Mesh]) OR (Extracorporeal Shockwave Therapies) OR (Shockwave Therapies, Extracorporeal) OR (Shockwave Therapy, Extracorporeal) OR (Therapy, Extracorporeal Shockwave) OR (Extracorporeal Shock Wave Therapy) OR (Shock Wave Therapy) OR (Shock Wave Therapies) OR (Therapy, Shock Wave) OR (Extracorporeal High-Intensity Focused Ultrasound Therapy) OR (Extracorporeal High Intensity Focused Ultrasound Therapy) OR (High-Intensity Focused Ultrasound Therapy) OR (High Intensity Focused Ultrasound Therapy) OR (HIFU Therapy) OR (HIFU Therapies) OR (Therapy, HIFU)) |
| Navicular syndrome | ((navicular) OR (sesamoid)) AND ((Horse) OR (Equus caballus) OR (Horse, Domestic) OR (Domestic Horse) OR (Domestic Horses) OR (Horses, Domestic) OR (Equus przewalskii) OR ("Horses"[Mesh])) AND (("Extracorporeal Shockwave Therapy"[Mesh]) OR (Extracorporeal Shockwave Therapies) OR (Shockwave Therapies, Extracorporeal) OR (Shockwave Therapy, Extracorporeal) OR (Therapy, Extracorporeal Shockwave) OR (Extracorporeal Shock Wave Therapy) OR (Shock Wave Therapy) OR (Shock Wave Therapies) OR (Therapy, Shock Wave) OR (Extracorporeal High-Intensity Focused Ultrasound Therapy) OR (Extracorporeal High Intensity Focused Ultrasound Therapy) OR (High-Intensity Focused Ultrasound Therapy) OR (High Intensity Focused Ultrasound Therapy) OR (HIFU Therapy) OR (HIFU Therapies) OR (Therapy, HIFU)) |
| Back pain syndrome | ((kissing spines)OR (back pain) OR (EBD) OR (thoracolumbar pain)) AND ((Horse) OR (Equus caballus) OR (Horse, Domestic) OR (Domestic Horse) OR (Domestic Horses) OR (Horses, Domestic) OR (Equus przewalskii) OR ("Horses"[Mesh])) AND (("Extracorporeal Shockwave Therapy"[Mesh]) OR (Extracorporeal Shockwave Therapies) OR (Shockwave Therapies, Extracorporeal) OR (Shockwave Therapy, Extracorporeal) OR (Therapy, Extracorporeal Shockwave) OR (Extracorporeal Shock Wave Therapy) OR (Shock Wave Therapy) OR (Shock Wave Therapies) OR (Therapy, Shock Wave) OR (Extracorporeal High-Intensity Focused Ultrasound Therapy) OR (Extracorporeal High Intensity Focused Ultrasound Therapy) OR (High-Intensity Focused Ultrasound Therapy) OR (High Intensity Focused Ultrasound Therapy) OR (HIFU Therapy) OR (HIFU Therapies) OR (Therapy, HIFU)) |
| Superficial digital flexor tendonitis | ((Tendinopathies) OR (Tendonopathy) OR (Tendonopathies) OR (Tendinitis) OR (Tendinitides) OR (Tendonitis) OR (Tendonitides) OR (Tendinosis) OR (Tendinoses) OR ("Tendinopathy"[Mesh])) AND ((Horse) OR (Equus caballus) OR (Horse, Domestic) OR (Domestic Horse) OR (Domestic Horses) OR (Horses, Domestic) OR (Equus przewalskii) OR ("Horses"[Mesh])) AND (("Extracorporeal Shockwave Therapy"[Mesh]) OR (Extracorporeal Shockwave Therapies) OR (Shockwave Therapies, Extracorporeal) OR (Shockwave Therapy, Extracorporeal) OR (Therapy, Extracorporeal Shockwave) OR (Extracorporeal Shock Wave Therapy) OR (Shock Wave Therapy) OR (Shock Wave Therapies) OR (Therapy, Shock Wave) OR (Extracorporeal High-Intensity Focused Ultrasound Therapy) OR (Extracorporeal High Intensity Focused Ultrasound Therapy) OR (High-Intensity Focused Ultrasound Therapy) OR (High Intensity Focused Ultrasound Therapy) OR (HIFU Therapy) OR (HIFU Therapies) OR (Therapy, HIFU)) |
